# Supplementary material for: Identification and functional analysis of non-coding regulatory small RNA FenSr3 in Bacillus amyloliquefaciens LPB-18
Source: PeerJ. 2023 May 15;11:e15236. doi: 10.7717/peerj.15236 (PMC10194069; doi:10.7717/peerj.15236)
Supplement: Supplemental Information 4 [file peerj-11-15236-s004.zip › KO/CK-vs-T1_map/map04152.html]

KEGG PATHWAY: AMPK signaling pathway - Reference pathway


|  |  |
| --- | --- |
| **AMPK signaling pathway - Reference pathway** |  |

[
Pathway menu
| Organism menu
| Pathway entry
| Show description
| User data mapping
]

AMP-activated protein kinase (AMPK) is a serine threonine kinase that is highly conserved through evolution. AMPK system acts as a sensor of cellular energy status. It is activated by increases in the cellular AMP:ATP ratio caused by metabolic stresses that either interfere with ATP production (eg, deprivation for glucose or oxygen) or that accelerate ATP consumption (eg, muscle contraction). Several upstream kinases, including liver kinase B1 (LKB1), calcium/calmodulin kinase kinase-beta (CaMKK beta), and TGF-beta-activated kinase-1 (TAK-1), can activate AMPK by phosphorylating a threonine residue on its catalytic alpha-subunit. Once activated, AMPK leads to a concomitant inhibition of energy-consuming biosynthetic pathways, such as protein, fatty acid and glycogen synthesis, and activation of ATP-producing catabolic pathways, such as fatty acid oxidation and glycolysis.

|  |  |  |
| --- | --- | --- |
| Reference pathway | 184% 150% 122% 100% 82% 67% 55% | 图片下载 |
